# Crystal structure of (2-chloro­eth­yl)[2-(methyl­sulfan­yl)benz­yl]ammonium chloride

**DOI:** 10.1107/S2056989015008221

**Published:** 2015-05-13

**Authors:** P. Raghavendra Kumar, Upereti Shailesh, B. S. Palakshamurthy

**Affiliations:** a Department of Chemistry, UCS, Tumkur University, Tumkur 572 103, Karnataka, India; b Department of Chemistry, Indian Institute of Technology, Delhi, New Delhi 110 016, India; c Department of Studies and Research in Physics, U.C.S., Tumkur University, Tumkur, Karnataka 572 103, India

**Keywords:** crystal structure, chloro­eth­yl, S and N donors, amine hydro­chloride, N—H⋯Cl hydrogen bonds

## Abstract

In the title mol­ecular salt, C_10_H_15_ClNS^+^·Cl^−^, the cation is [*R*′*R*"NH_2_]^+^, where *R*′ is 2-MeS-C_6_H_4_CH_2_– and *R*" is –CH_2_CH_2_Cl, and the anion is Cl^−^. In the cation, the N atom is protonated with *sp* ^3^-hybridization and with a tetra­hedral geometry. In the crystal, the anions are connected to the cations through two pairs of N—H⋯Cl hydrogen bonds, generating a four-centred inversion dimer with an *R*_4_^2^(8) ring motif.

## Abstract 2

In the cation of the title mol­ecular salt, the N atom is protonated with *sp* ^3^-hybridization and has a tetra­hedral geometry. In the crystal, the cations are bridged by the Cl^−^ anions *via* N—H⋯Cl hydrogen bonds, forming four-centred inversion dimers with an 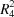
(8) ring motif.

## Chemical context   

Chloro­ethyl-functionalized derivatives containing S- and N-donor sites are used for the preparation of (S, N, S/Se/Te/P/As/Sb)-type tridentate hybrid ligands by nucleophilic substitution of the chloro (Cl^−^) group by *R*S^−^, ArSe^−^, ArTe^−^, Ph_2_P^−^, Ar_2_As^−^ (Kumar *et al.*, 2008*a* ▸; Singh *et al.*, 1999 ▸; Singh & Singh, 2010 ▸, 2012 ▸; Kumar *et al.*, 2008*b* ▸). Metal complexes of this type of hybrid ligand are important and have found applications as catalysts in organic synthesis (Singh *et al.*, 2013 ▸). Keeping this in mind, it was thought worthwhile to synthesise and characterise the title mol­ecular salt. We report herein on its synthesis, by chlorination of 2-(2-methyl­thio)­benzyl­amino)­ethanol using thionyl chloride, and on its crystal structure.
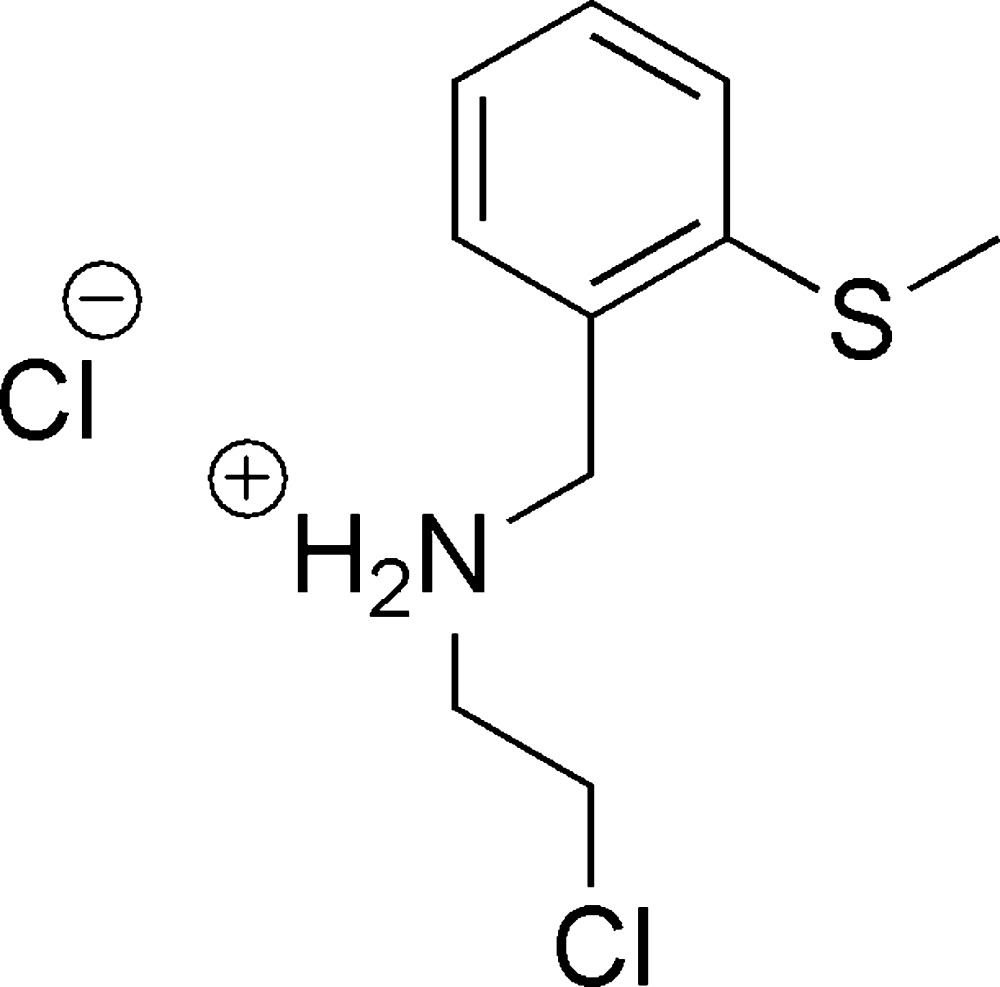


## Structural commentary   

**Figure 1:**
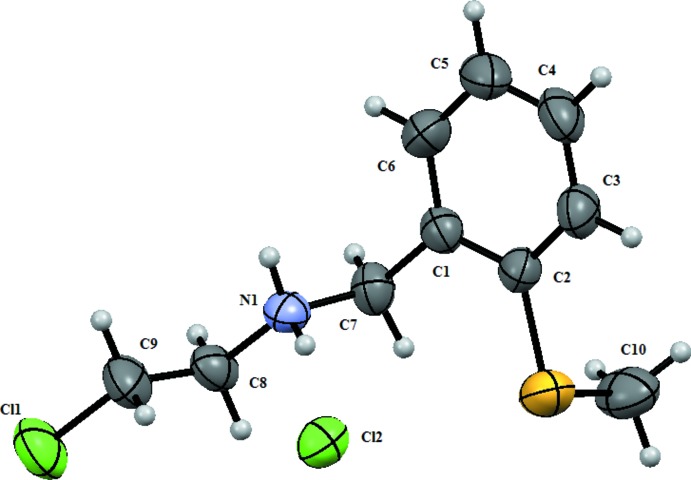
The mol­ecular structure of the title mol­ecular salt, showing the atom labelling. The displacement ellipsoids are drawn at the 50% probability level.

In the cation of the title mol­ecular salt (Fig. 1 ▸), the –CH_2_–N^+^H_2_–CH_2_–CH_2_–Cl substituent has an extended conformation with all of the non-H atoms lying in a plane [maximum deviation = 0.032 (4) Å for atom C8]. The N1 atom is protonated with *sp* ^3^-hybridization and has a tetra­hedral geometry. The S1 atom lies in the plane of the benzene ring to which it is attached while the methyl C10 atom is displaced from the plane of the benzene ring by 1.773 (5) Å.

The title mol­ecular salt was also characterised by NMR and FT–IR spectroscopy. In the proton NMR spectrum, the signals for the NCH_2_ and CH_2_Cl protons gave two triplets at 3.25 and 3.9 p.p.m., respectively. The [C_10_H_15_ClSN]^+^ cation is a secondary ammonium ion in which the N atom is protonated and hence undergoes *sp* ^3^ hybridization, resulting in a tetra­hedral geometry around the N atom. This was confirmed by NMR as the 〉NH_2_^+^ protons are highly deshielded and are observed as a broad singlet at 10.03 p.p.m. In the FT–IR spectrum of title salt, the N–H stretching band was observed at 1569 cm^−1^.

## Supra­molecular features   

**Figure 2:**
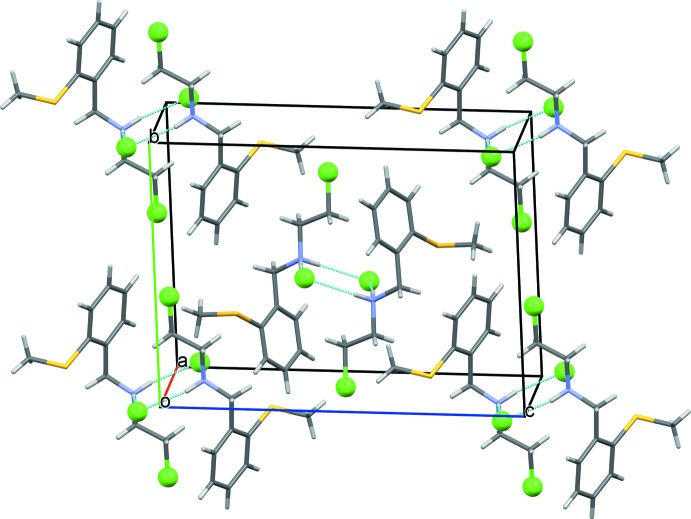
The crystal packing of the title mol­ecular salt, viewed along the *a* axis. The N—H⋯Cl hydrogen bonds are shown as dashed lines (see Table 1 ▸ for details).

In the crystal, the cation and anion are connected through two pairs of N—H⋯Cl hydrogen bonds. These hydrogen bonds result in the formation of four-centred inversion dimers with an 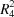
(8) ring motif (Table 1 ▸ and Fig. 2 ▸).

## Database survey   

A search of the Cambridge Structural Database (Version 5.36; Groom & Allen, 2014 ▸) found no hits for similar compounds. However, tridentate (S, N, S/Se/Te)-type ligands containing the cationic part of the title salt and their Pd^II^ and Ru^II^ complexes have been synthesised and structurally characterized (Kumar *et al.*, 2008*a* ▸; Singh & Singh, 2012 ▸; Singh *et al.*, 2012 ▸).

## Synthesis and crystallization   

**Figure 3:**
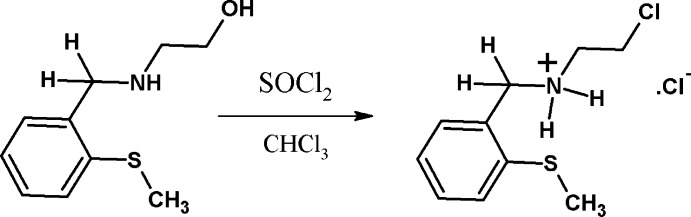
The synthesis of the title mol­ecular salt.

The synthesis of the title compound is illustrated in Fig. 3 ▸. 2-(2-Methyl­thio)­benzyl­amino)­ethanol (2 g, 10 mmol) was dissolved in 20 ml of dry chloro­form and the solution was cooled in an ice bath. Freshly distilled SOCl_2_ (3 ml, 40 mmol) dissolved in 20 ml of dry chloro­form was added to it dropwise over a period of 15 min. When the addition was complete, the temperature of the reaction mixture was increased slowly and the mixture was stirred under reflux for 6 h. Thereafter, the reaction mixture was cooled and concentrated to 10 ml on a rotary evaporator, giving a light-brown solid. The solid was dissolved in 10 ml of methanol, boiled with a pinch of activated charcoal and filtered. The filtrate was treated with 20 ml of diethyl ether. It gave a white crystalline product (caution: eye and skin irritant), which was filtered, washed with diethyl ether (10 ml × 4) and dried between the folds of filter paper. Colourless prisms of the title compound were grown in ethanol by slow evaporation of the solvent (yield: 70%; m.p.: 413 K; Λ_M_ = 3.0 cm^2^ mol^−1^ ohm^−1^. Elemental analysis, found (calc.): C, 47.87 (47.68), H, 5.95 (5.99), N, 5.68 (5.55) %; ^1^H NMR (CDCl_3_, 298 K): δ (*vs* TMS): 2.55 (*s*, 3H, SCH_3_), 3.25 (*t*, *J* = 6.09 Hz, 2H, H_1_), 3.9 (*t*, *J* = 6,6 Hz, 2H, H_2_), 4.94 (*s*, 2H, H_3_), 7.26 (*t*, *J* = 6.96 Hz, 1H, H_8_), 7.34–7.46 (*m*, 2H, H_6,7_), 7.72–7.74 (*d*, *J* = 7.5 Hz, 1H, H_9_), 10.03 (*bs*, 2H, NH_2_^+^). ^13^C{^1^H} NMR (CDCl_3_, 298 K): δ (*vs* TMS): 16.85 (SCH_3_), 48.17 (C_2_), 49.27 (C_1_), 57.12 (C_3_), 126.26 (C_6_), 127.89 (C_7_) , 128.87 (C_4_), 130.25 (C_8_), 131.50 (C_9_), 138.95 (C_5_). FT–IR (KBr, cm^−1^): 3415 (*s*), 1569 (*b*) (N–H), 1590 (C–N), 763 (C–S).

## Refinement   

Crystal data, data collection and structure refinement details are summarized in Table 2 ▸. The hydrogen atoms attached to atom N1 were located in a difference Fourier map. In the final cycles of refinement they were included in calculated positions, as were the C-bound H atoms, and treated as riding atoms: N—H = 0.89 Å, C—H = 0.93–0.97 Å with U_iso_(H) = 1.5*U*_eq_(C) for methyl H atoms and = 1.2*U*_eq_(N,C) for other H atoms.

## Supplementary Material

Crystal structure: contains datablock(s) I, Global. DOI: 10.1107/S2056989015008221/su5075sup1.cif

Structure factors: contains datablock(s) I. DOI: 10.1107/S2056989015008221/su5075Isup2.hkl

Supporting information file. DOI: 10.1107/S2056989015008221/su5075Isup3.cml

CCDC reference: 299500

Additional supporting information:  crystallographic information; 3D view; checkCIF report

## Figures and Tables

**Table 1:** Hydrogen-bond geometry (, )

*D*H*A*	*D*H	H*A*	*D* *A*	*D*H*A*
N1H1*B*Cl2	0.89	2.21	3.090(3)	169
N1H1*A*Cl2^i^	0.89	2.32	3.163(3)	158

Symmetry code: (i) 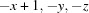.

**Table 2:** Experimental details

Crystal data	
Chemical formula	C_10_H_15_ClNS^+^Cl
*M*_r_	252.19
Crystal system, space group	Monoclinic, *P*2_1_/*n*
Temperature (K)	298
*a*, *b*, *c* ()	6.5717(10), 11.8058(17), 16.201(2)
()	97.374(3)
*V* (^3^)	1246.5(3)
*Z*	4
Radiation type	Mo *K*
(mm^1^)	0.65
Crystal size (mm)	0.28 0.24 0.20

Data collection	
Diffractometer	Bruker APEXII CCD
Absorption correction	Multi-scan (*SADABS*; Bruker, 2013 ▸)
*T*_min_, *T*_max_	0.839, 0.881
No. of measured, independent and observed [*I* > 2(*I*)] reflections	9002, 2255, 1584
*R*_int_	0.100
(sin /)_max_ (^1^)	0.600

Refinement	
*R*[*F* ^2^ > 2(*F* ^2^)], *wR*(*F* ^2^), *S*	0.065, 0.158, 1.04
No. of reflections	2255
No. of parameters	128
H-atom treatment	H-atom parameters constrained
_max_, _min_ (e ^3^)	0.50, 0.23

Computer programs: *APEX2* and *SAINT* (Bruker, 2013 ▸), *SHELXS97* (Sheldrick, 2008 ▸), *SHELXL2014* (Sheldrick, 2015 ▸), *Mercury* (Macrae *et al.*, 2008 ▸) and *PLATON* (Spek, 2009 ▸).

## supplementary crystallographic information

### Crystal data

**Table:**

C_10_H_15_ClNS^+^·Cl^−^	*F*(000) = 528
*M**_r_* = 252.19	*D*_x_ = 1.344 Mg m^−^^3^
Monoclinic, *P*2_1_/*n*	Mo *K*α radiation, λ = 0.71073 Å
*a* = 6.5717 (10) Å	θ = 2.1–25.0°
*b* = 11.8058 (17) Å	µ = 0.65 mm^−^^1^
*c* = 16.201 (2) Å	*T* = 298 K
β = 97.374 (3)°	Prism, colourless
*V* = 1246.5 (3) Å^3^	0.28 × 0.24 × 0.20 mm
*Z* = 4	

### Data collection

**Table:**

Bruker APEXII CCD diffractometer	1584 reflections with *I* > 2σ(*I*)
Radiation source: fine-focus sealed tube	*R*_int_ = 0.100
phi and ω scans	θ_max_ = 25.3°, θ_min_ = 2.1°
Absorption correction: multi-scan (*SADABS*; Bruker, 2013)	*h* = −7→7
*T*_min_ = 0.839, *T*_max_ = 0.881	*k* = −14→14
9002 measured reflections	*l* = −19→19
2255 independent reflections	

### Refinement

**Table:**

Refinement on *F*^2^	0 restraints
Least-squares matrix: full	Hydrogen site location: inferred from neighbouring sites
*R*[*F*^2^ > 2σ(*F*^2^)] = 0.065	H-atom parameters constrained
*wR*(*F*^2^) = 0.158	*w* = 1/[σ^2^(*F*_o_^2^) + (0.0727*P*)^2^] where *P* = (*F*_o_^2^ + 2*F*_c_^2^)/3
*S* = 1.04	(Δ/σ)_max_ < 0.001
2255 reflections	Δρ_max_ = 0.50 e Å^−^^3^
128 parameters	Δρ_min_ = −0.23 e Å^−^^3^

### Special details

**Table:**

Geometry. All e.s.d.'s (except the e.s.d. in the dihedral angle between two l.s. planes) are estimated using the full covariance matrix. The cell e.s.d.'s are taken into account individually in the estimation of e.s.d.'s in distances, angles and torsion angles; correlations between e.s.d.'s in cell parameters are only used when they are defined by crystal symmetry. An approximate (isotropic) treatment of cell e.s.d.'s is used for estimating e.s.d.'s involving l.s. planes.

### Fractional atomic coordinates and isotropic or equivalent isotropic displacement parameters (Å^2^)

**Table:**

	*x*	*y*	*z*	*U*_iso_*/*U*_eq_	
S1	0.73384 (17)	−0.10230 (10)	0.26820 (8)	0.0632 (4)	
Cl1	0.2925 (2)	0.37387 (9)	0.02280 (9)	0.0769 (4)	
N1	0.3518 (5)	0.0518 (2)	0.10476 (19)	0.0437 (8)	
H1A	0.2751	0.0208	0.0613	0.052*	
H1B	0.4827	0.0411	0.0980	0.052*	
C1	0.3504 (6)	−0.1317 (3)	0.1757 (2)	0.0442 (9)	
C2	0.5336 (6)	−0.1815 (3)	0.2109 (2)	0.0443 (9)	
C4	0.4120 (7)	−0.3622 (4)	0.1571 (3)	0.0615 (12)	
H4	0.4340	−0.4393	0.1503	0.074*	
C3	0.5612 (7)	−0.2969 (3)	0.2011 (3)	0.0557 (11)	
H3	0.6828	−0.3307	0.2247	0.067*	
C5	0.2319 (8)	−0.3142 (4)	0.1233 (3)	0.0675 (13)	
H5	0.1301	−0.3584	0.0940	0.081*	
C6	0.2013 (7)	−0.1992 (4)	0.1330 (3)	0.0612 (12)	
H6	0.0777	−0.1669	0.1102	0.073*	
C8	0.3102 (6)	0.1754 (3)	0.1051 (3)	0.0497 (10)	
H8A	0.4013	0.2113	0.1493	0.060*	
H8B	0.1701	0.1884	0.1157	0.060*	
C7	0.3103 (6)	−0.0073 (3)	0.1812 (2)	0.0489 (10)	
H7A	0.1683	0.0047	0.1896	0.059*	
H7B	0.3968	0.0241	0.2287	0.059*	
C9	0.3421 (8)	0.2266 (3)	0.0230 (3)	0.0657 (13)	
H9A	0.2511	0.1906	−0.0212	0.079*	
H9B	0.4822	0.2136	0.0125	0.079*	
C10	0.6669 (8)	−0.1210 (4)	0.3711 (3)	0.0762 (15)	
H10A	0.5336	−0.0892	0.3741	0.114*	
H10B	0.7662	−0.0834	0.4104	0.114*	
H10C	0.6651	−0.2003	0.3840	0.114*	
Cl2	0.80532 (15)	0.04958 (9)	0.07270 (6)	0.0537 (3)	

### Atomic displacement parameters (Å^2^)

**Table:**

	*U*^11^	*U*^22^	*U*^33^	*U*^12^	*U*^13^	*U*^23^
S1	0.0557 (7)	0.0679 (8)	0.0632 (8)	−0.0149 (6)	−0.0034 (6)	0.0005 (6)
Cl1	0.0879 (9)	0.0413 (7)	0.1012 (10)	0.0074 (6)	0.0111 (7)	0.0136 (6)
N1	0.0465 (19)	0.0362 (18)	0.0467 (19)	0.0002 (14)	−0.0010 (15)	−0.0050 (15)
C1	0.053 (2)	0.038 (2)	0.042 (2)	0.0005 (19)	0.0068 (19)	0.0023 (17)
C2	0.050 (2)	0.041 (2)	0.040 (2)	0.0010 (18)	−0.0013 (18)	0.0014 (18)
C4	0.088 (4)	0.035 (2)	0.060 (3)	0.005 (2)	0.005 (3)	0.005 (2)
C3	0.064 (3)	0.042 (2)	0.058 (3)	0.011 (2)	−0.003 (2)	0.004 (2)
C5	0.088 (4)	0.050 (3)	0.058 (3)	−0.018 (3)	−0.011 (3)	0.001 (2)
C6	0.054 (3)	0.058 (3)	0.066 (3)	−0.005 (2)	−0.013 (2)	0.005 (2)
C8	0.049 (2)	0.032 (2)	0.067 (3)	0.0023 (18)	0.006 (2)	−0.001 (2)
C7	0.056 (2)	0.043 (2)	0.048 (2)	0.0082 (19)	0.007 (2)	0.0038 (18)
C9	0.085 (3)	0.037 (2)	0.071 (3)	0.002 (2)	−0.003 (3)	0.004 (2)
C10	0.084 (4)	0.084 (4)	0.058 (3)	−0.020 (3)	−0.001 (3)	−0.014 (3)
Cl2	0.0499 (6)	0.0577 (7)	0.0524 (6)	0.0044 (5)	0.0020 (5)	−0.0074 (5)

### Geometric parameters (Å, º)

**Table:**

S1—C2	1.776 (4)	C3—H3	0.9300
S1—C10	1.792 (5)	C5—C6	1.384 (6)
Cl1—C9	1.768 (4)	C5—H5	0.9300
N1—C7	1.477 (5)	C6—H6	0.9300
N1—C8	1.485 (5)	C8—C9	1.501 (6)
N1—H1A	0.8900	C8—H8A	0.9700
N1—H1B	0.8900	C8—H8B	0.9700
C1—C6	1.378 (5)	C7—H7A	0.9700
C1—C2	1.394 (5)	C7—H7B	0.9700
C1—C7	1.496 (5)	C9—H9A	0.9700
C2—C3	1.386 (5)	C9—H9B	0.9700
C4—C5	1.362 (6)	C10—H10A	0.9600
C4—C3	1.373 (6)	C10—H10B	0.9600
C4—H4	0.9300	C10—H10C	0.9600
			
C2—S1—C10	99.7 (2)	N1—C8—C9	110.2 (3)
C7—N1—C8	114.0 (3)	N1—C8—H8A	109.6
C7—N1—H1A	108.8	C9—C8—H8A	109.6
C8—N1—H1A	108.8	N1—C8—H8B	109.6
C7—N1—H1B	108.8	C9—C8—H8B	109.6
C8—N1—H1B	108.8	H8A—C8—H8B	108.1
H1A—N1—H1B	107.6	N1—C7—C1	111.1 (3)
C6—C1—C2	118.8 (4)	N1—C7—H7A	109.4
C6—C1—C7	118.6 (4)	C1—C7—H7A	109.4
C2—C1—C7	122.6 (3)	N1—C7—H7B	109.4
C3—C2—C1	119.1 (4)	C1—C7—H7B	109.4
C3—C2—S1	118.6 (3)	H7A—C7—H7B	108.0
C1—C2—S1	122.3 (3)	C8—C9—Cl1	110.5 (3)
C5—C4—C3	120.1 (4)	C8—C9—H9A	109.5
C5—C4—H4	120.0	Cl1—C9—H9A	109.5
C3—C4—H4	120.0	C8—C9—H9B	109.5
C4—C3—C2	121.1 (4)	Cl1—C9—H9B	109.5
C4—C3—H3	119.5	H9A—C9—H9B	108.1
C2—C3—H3	119.5	S1—C10—H10A	109.5
C4—C5—C6	119.6 (4)	S1—C10—H10B	109.5
C4—C5—H5	120.2	H10A—C10—H10B	109.5
C6—C5—H5	120.2	S1—C10—H10C	109.5
C1—C6—C5	121.3 (4)	H10A—C10—H10C	109.5
C1—C6—H6	119.3	H10B—C10—H10C	109.5
C5—C6—H6	119.3		
			
C6—C1—C2—C3	0.7 (6)	C3—C4—C5—C6	0.6 (7)
C7—C1—C2—C3	−178.2 (4)	C2—C1—C6—C5	−1.2 (6)
C6—C1—C2—S1	−179.2 (3)	C7—C1—C6—C5	177.8 (4)
C7—C1—C2—S1	1.9 (5)	C4—C5—C6—C1	0.5 (7)
C10—S1—C2—C3	−87.1 (4)	C7—N1—C8—C9	−175.9 (3)
C10—S1—C2—C1	92.8 (4)	C8—N1—C7—C1	178.6 (3)
C5—C4—C3—C2	−1.1 (7)	C6—C1—C7—N1	−81.3 (5)
C1—C2—C3—C4	0.4 (6)	C2—C1—C7—N1	97.7 (4)
S1—C2—C3—C4	−179.7 (3)	N1—C8—C9—Cl1	179.9 (3)

### Hydrogen-bond geometry (Å, º)

**Table:**

*D*—H···*A*	*D*—H	H···*A*	*D*···*A*	*D*—H···*A*
N1—H1*B*···Cl2	0.89	2.21	3.090 (3)	169
N1—H1*A*···Cl2^i^	0.89	2.32	3.163 (3)	158

Symmetry code: (i) −*x*+1, −*y*, −*z*.
